# *Helicobacter bizzozeronii* infection in a girl with severe gastric disorders in México: case report

**DOI:** 10.1186/s12887-023-04142-7

**Published:** 2023-07-15

**Authors:** Ericka Montijo-Barrios, Omaha Y. Celestino-Pérez, Luis Morelia-Mandujano, Cesar Mauricio Rojas-Maruri, Annemieke Smet, Freddy Haesebrouck, Chloë De Witte, Carolina Romo-González

**Affiliations:** 1 Pediatric Gastroenterology and Nutrition Service, National Institute of Pediatrics, Mexico City, Mexico; 2 Laboratory of Experimental Bacteriology, National Institute of Pediatrics, Mexico City, Mexico; 3 Surgical Pathology Service, National Institute of Pediatrics, Mexico City, Mexico; 4 Translational Research in Immunology and Inflammation, Laboratory of Experimental Medicine and Pediatrics, Faculty of Medicine and Health Sciences, University of Antwerp, Wilrijk, Belgium; 5 Department of Pathobiology, Pharmacology and Zoological Medicine, Faculty of Veterinary Medicine, Ghent University, Ghent, Belgium

**Keywords:** *H. bizzozeronii*, *H. pylori*, Epigastric pain, Gastric ulcer, Case report

## Abstract

**Background:**

Gastric non-*Helicobacter pylori* helicobacters (NHPH) naturally colonize the stomach of animals. In humans, infection with these bacteria is associated with chronic active gastritis, peptic ulceration and MALT-lymphoma. *H. bizzozeronii* belongs to these NHPH and its prevalence in children is unknown.

**Case presentation:**

This case report describes for the first time a NHPH infection in a 20-month-old girl with severe gastric disorders in Mexico. The patient suffered from melena, epigastric pain, and bloating. Gastroscopy showed presence of a Hiatus Hill grade I, a hemorrhagic gastropathy in the fundus and gastric body, and a Forrest class III ulcer in the fundus. Histopathologic examination revealed a chronic active gastritis with presence of long, spiral-shaped bacilli in the glandular lumen. Biopsies from antrum, body and incisure were negative for presence of *H. pylori* by culture and PCR, while all biopsies were positive for presence of *H. bizzozeronii* by PCR. Most likely, infection occurred through intense contact with the family dog. The patient received a triple therapy consisting of a proton pump inhibitor, clarithromycin, and amoxicillin for 14 days, completed with sucralfate for 6 weeks, resulting in the disappearance of her complaints.

**Conclusion:**

The eradication could not be confirmed, although it was suggested by clear improvement of symptoms. This case report further emphasizes the zoonotic importance of NHPH. It can be advised to routinely check for presence of both *H. pylori* and NHPH in human patients with gastric complains.

## Background

*Helicobacter* spp. are Gram-negative, motile bacteria which colonize the gastro-intestinal tract of humans and animals. *H. pylori* is the best-studied and most prevalent *Helicobacter* species colonizing the human stomach. Infection with *H. pylori* has been associated with severe gastric disorders, such as peptic ulceration, mucosa-associated lymphoid tissue (MALT)-lymphoma, and/or adenocarcinoma [1].

Apart from *H. pylori*, spiral-shaped non-*H. pylori* Helicobacters (NHPHs) have been demonstrated in 0.2–12% of human patients with gastric complaints undergoing gastric biopsy [2]. Infection with these bacteria has been associated with the development of chronic active gastritis, peptic ulceration, and MALT-lymphoma [3, 4]. The risk for developing MALT-lymphoma is higher during NHPH infection compared to *H. pylori* infection, although the induced gastritis may be less severe. Co-infections with *H. pylori* have also been described and have been associated with an increased incidence of peptic ulceration [4–7]. So far, detected NHPH in the human stomach are *H. suis, H. felis, H. bizzozeronii, H. salomonis*, and *H. heilmannii* [8–14]. *H. suis* naturally colonizes the stomach of pigs and non-human primates, while *H. felis, H. bizzozeronii, H. salomonis*, and *H. heilmannii* are associated with dogs and cats. Living in proximity as well as intense contact with infected animals have been suggested to be a risk factor for humans to contract NHPH infection.

Unlike *H. pylori*, commercial noninvasive tests are not available for diagnosis of NHPH infections and gastric biopsy samples are not checked routinely for the presence of these bacteria, resulting in a potential underestimation of their prevalence. Furthermore, detection is often hampered by the patchy and sparse colonization pattern of NHPH. The epidemiology and pathology of gastric NHPH infections in human patients is unclear and remains to be further elucidated [15].

This case report describes for the first time a NHPH infection in a young girl suffering from severe gastric disorders in Mexico. Species identification raveled it to be *H. bizzozeronni*. Eradication was successful using standard triple therapy resulting in the dissapereance of her complaints.

## Narrative

### Anamnesis

A 20-month-old girl with gastric disorders was admitted to the emergency unit of the National Institute of Pediatrics, Mexico. The complaints started 3 weeks before. She suffered from upper gastrointestinal bleeding indicated by the presence of 3 episodes in one day of moderate melena. She also showed mild to moderate epigastric pain and bloating on a daily basis which was not related to certain kinds of food or eating. No treatment had been administred. There was no history of illness or gastro-intestinal problems. She lived together with her parents and 2 uncles in a small place with poor hygienic conditions and low sociocultural status. A dog had been living with them in the last 4 years and was allowed to defecate in the house. The girl had close and frequent contact with the family dog. The dog was correctly vaccinated and showed no clinical symptoms.

### Physical examination, blood analysis and gastroscopy

**Fig. 1:**
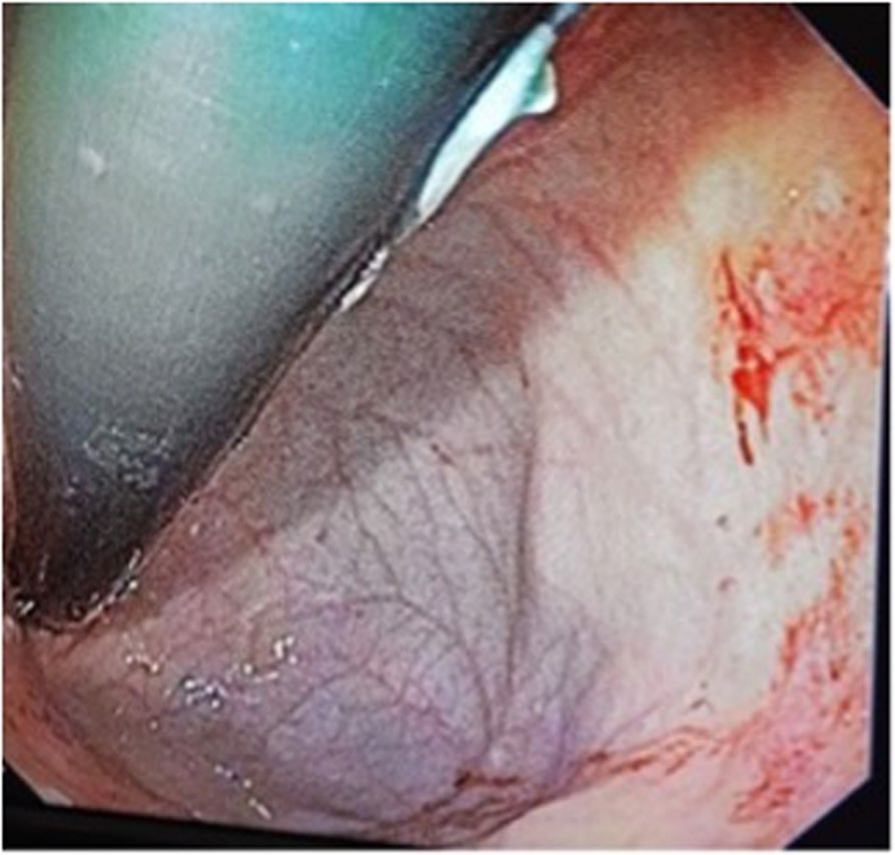
Gastroscopy of the patient reveals presence of hemorrhagic gastropathy in the fundic gland zone

Physical examination showed a high resting heart rate of 145 bpm, a well perfused circulation and a normal blood pressure. Abdominal examination yielded epigastric tenderness. The rest of the abdomen was soft with normal bowel sounds and there was no indication of organomegaly or masses. Complete blood count indicated presence of anaemia (Hb 4.2) with normocytic red blood cells. Liver function and coagulation tests were normal. Gastroscopy showed presence of a Hiatus Hill grade I, a hemorrhagic gastropathy in the fundus (Fig. 1) and gastric body, and a Forrest III ulcer in the fundus according to scoring system for ulcer Forrest Score [16]. Biopsies were taken from the gastric antrum, corpus and incisura according to Sidney protocol ASGE Standars of Practice Committee [17] these samples were sent for culture, histopathology, and PCR.

### Histopathology and PCR

**Fig. 2:**
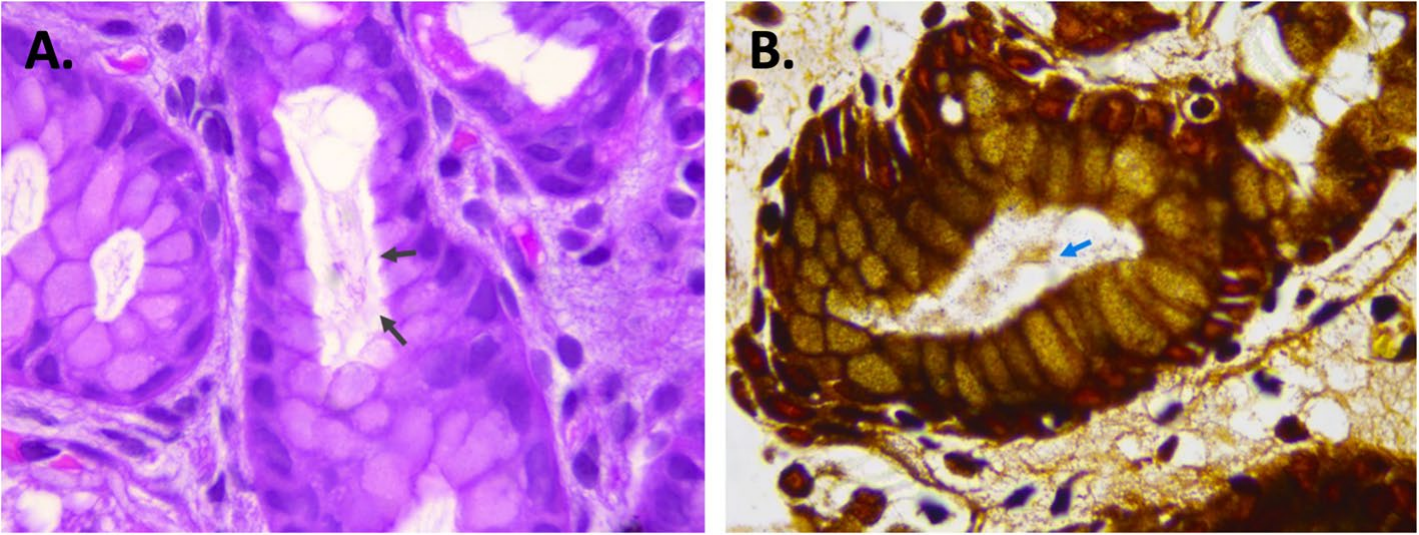
Hematoxylin-eosin (**A**) and Warthin Starry (**B**) staining of the patient’s antrum biopsy shows presence of large, spiral-shaped microorganisms located in the glandular lumen (black and blue arrows) (100X)

Gastric biopsy samples were fixed in 10% formalin, embedded in paraffin after which 5-µm sections were stained with hematoxylin/eosin (H&E) and Warthin Starry. Histopathologic examination showed presence of a mild interstitial chronic gastritis with mononuclear infiltrate (lymphocytes) in the fundus. The gastric corpus showed presence of a mild follicular (lymphocytes) and active (neutrophils) gastritis with presence of large, spiral-shaped bacteria in the glandular lumen both on H&E and Warthin-Starry staining (Fig. 2).

DNA was extracted from gastric biopsies using Wizard® Genomic DNA Purification Kit (Promega Corporation) according to the instructions of the manufacturer. These DNA extractions were subjected to a PCR to detect the presence of *H. pylori* and NHPH (i.e. *H. suis*, *H. heilmannii/H. ailurogastricus*, *H. felis*, *H. bizzozeronii*, and *H. salomonis*).

To identify *H. pylori* we performed culture and PCR. A part of the homogenization of each biopsy was cultured in Blood Agar Base (Becton Dickinson, Baltimore, MD, USA) supplemented with 10% sheep blood. The plates were grown at 37 °C under microaerophilic conditions [18]. For *H. pylori-*specific PCR part of the *ureC* glmM and *16 S rRNA* gene were amplified as reported [19, 20]. Culture and PCR were negative, indicating that the patient was not infected with *H. pylori*.

**Fig. 3:**
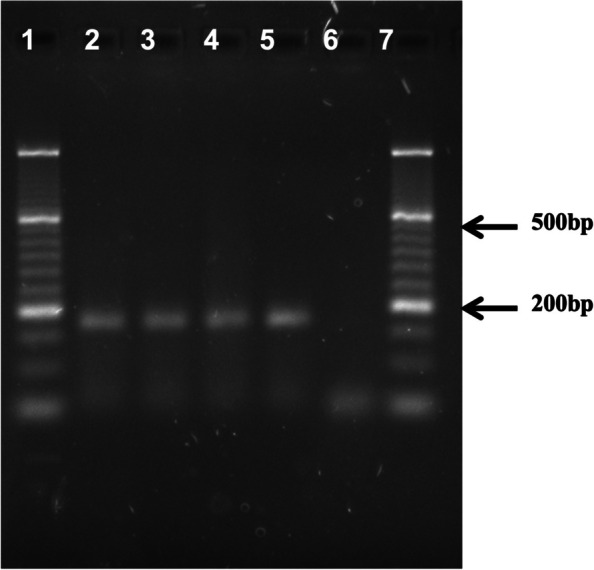
Analysis of 2.5% agarose gel. 500 bp DNA lader (lanes 1 and 7); antrum, corpus and incisure DNA (lanes 2 to 4); *H. bizozeronii* DNA, positive control (lane 5). An amplicon of 172 bp *H. bizozeronii* was observed

**Fig. 4:**
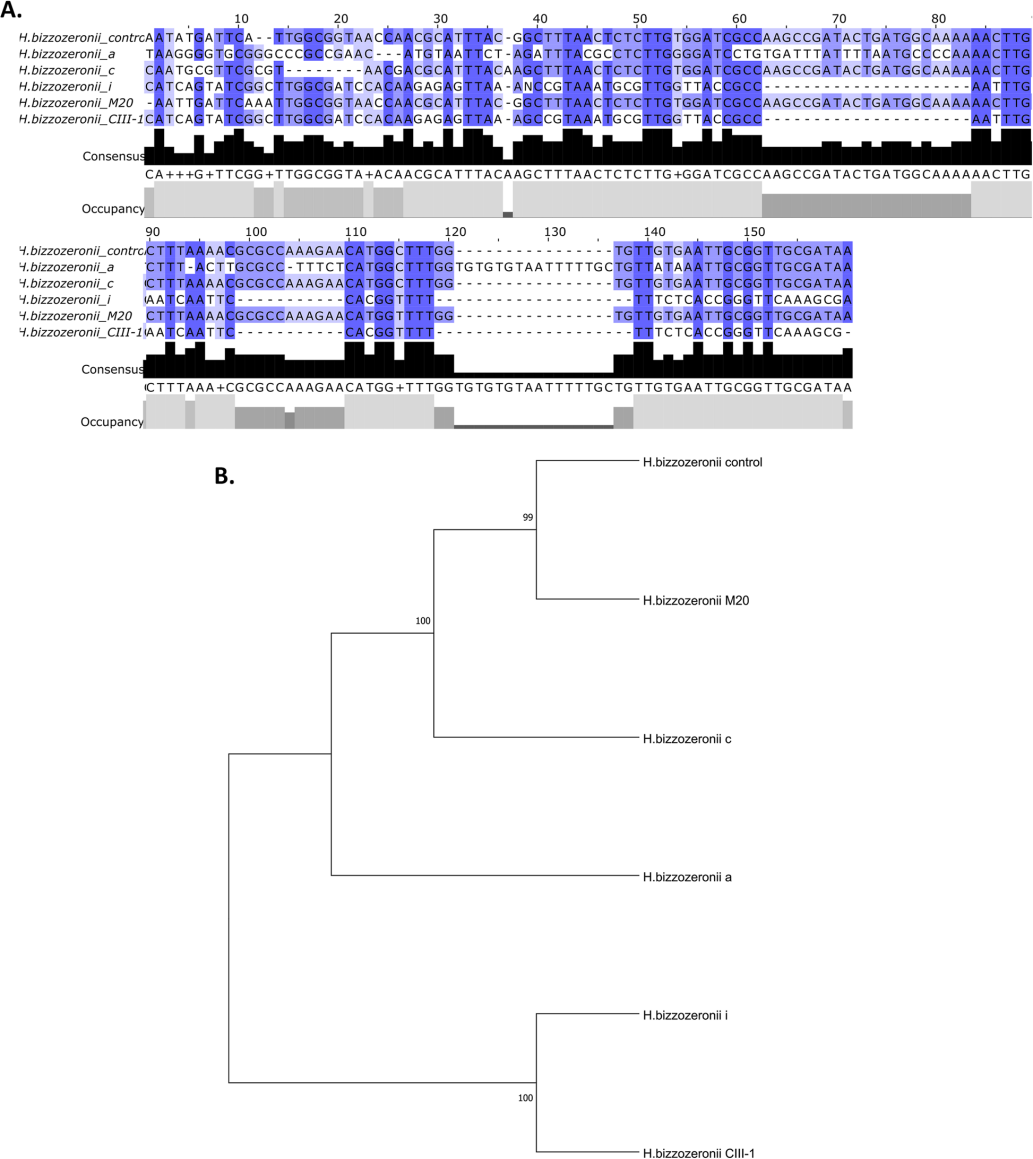
**A** Sequence alignments based on *H. bizzozeronii* urea gene, amplified from a control strain, and different stomach regions from case; reference sequence from *H. bizzozeroni*i CIII-1 and M20. **B** Phylogenetic tree, the numbers at the nodes indicate the level of bootstrap support (%) based on UPGMA method analyses of 500 bootstrap replications, and percentages are indicated on nodes, the distances were computed with the maximum composite likelihood method

For NHPH, part of the *ureA* gene was amplified using genus-and species-specific primers [7]. The thermal cycle program consisted of 95 °C for 15 min, followed by 40 cycles of denaturation at 95 °C for 20 s, annealing/extension at 60 °C for 30 s and elongation at 72 °C for 30 s. Biopsies from the gastric body, incisura and antrum tested positive for presence of *H. bizzozeronnii* DNA, corresponding to an amplification product of 172 bp (Fig. 3). To confirm the presence of gastric NHPH DNA, the PCR products were cleaned using the QIAquick Gel Extraction Kit (QIAGEN, Hilden, Germany) and nucleotide sequencing was performed using the dideoxynucleotide chain termination method with a BigDye Terminator v3.1 cycle sequencing kit in an ABI PRISM 377 automated DNA sequencer (Applied Biosystems, California, USA). The obtained sequence was compared with the reference sequences NCBI Reference Sequence The nucleotide sequences were analyzed by aligned with MEGA X program and the MUSCLE aligner were used with the UPGMA method. To view the alignments, we used the Jalview 2.11.1.4 program. Images of the multiple alignments were created, where the gaps, the alignment positions every 10 bases, and a consensus graph were marked with dashes. The sequences of the PCR products showed the highest alignment hit with the reference sequence NC_015674 (strain C-III-1) and NZ_FZLB01000041.1. Relationships of the evolutionary history of the taxa were inferred using the UPGMA method. The evolutionary distances were calculated using the compound maximum probability method and the optimal tree is shown in Fig. 4. The data presented in this study are deposited in the GenBank repository; accession number OM327339, OM327340, OM327341.

## Patient perspective

### Treatment and re-evaluation

The patient received blood transfusion to treat the anaemia, upon which her hemodynamic stability improved, tachycardia disappeared, and hemoglobin levels restored. To eradicate *H. bizzozeronnii*, the patient was orally treated with a triple therapy for 14 days (1 mg/kg omeoprazole, 20 mg/kg clarithromycin, and 90 mg/kg amoxicilin, once a day) in combination with sucralfate (80 mg/kg, once a day) for 6 weeks. The treatment was well tolerated and resulted in the disappearance of her complaints.

### Discussion and conclusion

Gastric NHPH are highly present in dogs and cats, with prevalences ranging from 60 up to 100% [21]. Evidence is accumulating that pets may serve as reservoir hosts for zoonotic NHPH [3]. In several case studies, transmission of NHPH was expected to occur from dogs and cats to their owner [8, 22–27]. Some studies even demonstrated presence of the same gastric species in the owner’s pets [22, 23, 28] further supporting the zoonotic potential of NHPH. Although we did not investigate presence of *H. bizzozeronnii* in the family dog, the patient most likely contracted infection through intense and close contact with the dog. Furthermore, *H. bizzozeronnii* is the most prevalent gastric *Helicobacter* species in dogs [29–31], which further strengthens this hypothesis.

Nevertheless, in most case studies the specific NHPH species was not determined. One study described the isolation of *H. bizzozeronnii* from the human gastric mucosa, highlighting its zoonotic potential [26, 27]. Van den Bulck et al. demonstrated that *H. suis* was the most prevalent NHPH species in human patients undergoing gastric biopsy (i.e. 37% of NHPH infected patients), followed by *H. salomonis* (21%), *H. felis* (15%), *H. heilmannii* (8%), and *H. bizzozeronnii* (4%). The low prevalence of *H. bizzozeronnii* in these human gastric biopsies seems to be in contrast with its predominance in the canine stomach. This might be related to differences in colonization ability between gastric *Helicobacter* spp, for example in their ability to adhere to the human gastric mucosa [32].

The exact route of NHPH transmission from animals to humans is not yet clears [3]. Several studies showed that living in close proximity to as well as intense contact with infected dogs, cats, and pigs leads to a significant risk of NHPH infection [33, 34]. *Helicobacter* DNA has been detected in saliva and faeces from cats and dogs [35], indicating that oral-oral and oral-faecal contact may be a route of transmission. In this case report, the dog was allowed to defecate in the house, which may have served as source of infection. Nevertheless, not all NHPH infected humans’ patients had contact with domesticated animals [8, 24]. An additional transmission route might be contaminated water, as *Helicobacter* spp. are able to survive in water for several hours [36]. Finally, the role of wild mice as vector might be considered as well, as rodents are easily colonized by most NHPH [3].

In humans, NHPH infections have been associated with a specific clinical sign like abdominal and/or epigastric pain, bloating, nausea, vomiting, dehydration, hematemesis, pyrosis, and dysphagia [27]. On gastroscopy, chronic active gastritis, nodular gastritis, erythema, ulceration and/or MALT-lymphoma may be present [8, 10, 12, 24, 37, 38]. Similarly, our patient showed signs of gastric disorders, as well as chronic active gastritis, mucosal erythema, and ulceration. It could be argued that these associations are incidental, in view of the low numbers of patients infected with NHPH. Nevertheless, experimental rodent models have shown that NHPH induce severe gastritis and MALT-lymphoma [23, 39]. In our and other studies, both clinical signs as well as gastric anomalies resolved after clearance of NHPH infection, further underlying the causal relationship.

Treatment of NHPH infections in humans is difficult to determine due to the lack of randomized trials [3]. In practice, treatment schemes successful in eradicating *H. pylori* are used. Triple therapy consisting of a proton pump inhibitor (omeprazole or pantoprazole) and 2 antibiotics (clarithromycin + amoxcillin or metronidazole) has already been shown to be effective, similar to this case report [40–42]. In some cases, NHPH were eradicated from the owner’s pet as well [23]. Here, treatment of the dog in combination with improvement of hygienic standards may be considered as well to prevent reinfection. Furthermore, follow-up endoscopy 6–12 months after treatment would be suitable to confirm complete clearance of *H. bizzozeronii* infection. The eradication could not be confirmed at this moment, although it was suggested by improvement of symptoms. Still, NHPH / Hp infections remain asymptomatic in > 80% of the patients having such gastric colonization. An urea breath test will not confirm the erradication, as it is often falsely negative in patients with NHPH.

Blaecher et al. showed a higher frequency of *H. suis* in human patients after *H. pylori* eradication, indicating that disappearance of *H. pylori* may create a niche for colonization of the human stomach by other pathogens, like NHPH. It can be advised to check for presence of these bacteria, especially when the patient shows persistent complaints in absence of *H. pylori*. Presence of NHPH can be implemented by histopathological analysis and/or by PCR on gastric biopsies.

In conclusion, for the first time, presence of a *H. bizzozeronnii* infection was shown in a young girl with gastric disorders in Mexico. This case report further emphasizes the zoonotic importance of NHPH. It can be advised to routinely check for presence of both *H. pylori* and NHPH in human patients with gastric complains.

## Data Availability

Data on patient and case details are available from the author on reasonable request.
